# Concordance Rates of Birth Defects After Assisted Reproductive Technology Among 17 258 Japanese Twin Pregnancies: A Nationwide Survey, 2004–2009

**DOI:** 10.2188/jea.JE20120103

**Published:** 2013-01-05

**Authors:** Syuichi Ooki

**Affiliations:** Department of Health Science, Ishikawa Prefectural Nursing University, Kahoku, Ishikawa, Japan; 石川県立看護大学健康科学講座

**Keywords:** birth defects, assisted reproductive technology (ART), twin pairs, concordance rate, nationwide epidemiologic study

## Abstract

**Background:**

Most twins after assisted reproductive technology (ART) are dizygotic. Analysis of dizygotic twin pairs is useful in assessing familial aggregation in the development of birth defects.

**Methods:**

Using nationwide post-ART data from the Japan Society of Obstetrics and Gynecology, recurrence risk ratios (RRRs)—defined as probandwise concordance rates of birth defects in twins divided by the prevalence of birth defects in the general population—were calculated as indicators of familial aggregation. Birth defects were then reclassified according to the ICD-10 categories corresponding to codes Q00–Q99. From 2004 to 2009, there were 17 258 twin pregnancies.

**Results:**

At least 1 birth defect was noted in 236 twin pairs: 11 concordant and 225 discordant pairs. Regarding major organ systems, high probandwise concordance rates were observed for congenital malformations of eye, ear, face, and neck (11.8%), cleft lip and cleft palate (10.5%), congenital malformations of the nervous system (9.8%), and other congenital malformations of the digestive system (9.5%). High RRRs were observed for congenital malformations of eye, ear, face, and neck (RRR = 233), specifically other congenital malformations of the ear (RRR = 449); congenital malformations of the great arteries (RRR = 235), specifically those of the patent ductus arteriosus (RRR = 530); and for cleft lip and cleft palate (RRR = 208), specifically cleft palate with cleft lip (RRR = 609). The probandwise concordance rate of any birth defect (8.9%) was nearly identical to the approximated recurrence risk of sib-pairs (8.8%), which assumed multifactorial inheritance.

**Conclusions:**

The present findings suggest that familial aggregation is a factor in some birth defects.

## Abstract (ja)

**【背景】:**

生殖補助医療によるふたごの大部分は二卵性である。二卵性ふたごペアの分析は、先天異常の形成過程における家族集積性を検討するうえで有効である。

**【方法】:**

日本産科婦人科学会が公表している全国的な生殖補助医療データを利用して、家族集積性の指標として再発危険率（RRR）を算出した。再発危険率はふたごペアにおける先天異常の発端者一致率を一般集団における先天異常の発生頻度で除した値と定義した。国際疾病分類第10版（ICD-10）のコードQ00-Q99（先天奇形、変形及び染色体異常）に従って、先天異常の症例データを再分類した。2004年から2009年までに17258件のふたご妊娠があった。

**【結果】:**

236組のペアに少なくとも1児の先天異常が認められた。一致が11組、不一致225組であった。主要組織分類に関しては、眼・耳・顔面及び頚部の先天奇形（11.8%）、唇裂及び口蓋裂（10.5%）、神経系の先天奇形（9.8%）、消化器系のその他の先天奇形（9.5%）で高い発端者一致率が認められた。眼・耳・顔面及び頚部の先天奇形（RRR=233）、特に、耳のその他の先天奇形（RRR=449）、大型動脈の先天奇形（RRR=235）、特に、動脈管開存症（RRR=530）、唇裂及び口蓋裂（RRR=208）、特に、唇裂を伴う口蓋裂（RRR=609）で高い再発危険率を認めた。先天異常全体の発端者一致率（8.9%）は、多因子遺伝を仮定して推定した同胞再発危険率（8.8%）とほぼ一致した。

**【結論】:**

今回の知見は、ある種の先天異常に家族集積性があることを示唆する。

## INTRODUCTION

According to data on assisted reproductive technology (ART) and vital statistics in Japan, the percentage of ART live births was 2.49% (26 680/1 070 035) in 2009, which indicates that ART is becoming widespread in Japan.^1^ To date, most population-based epidemiologic studies of twinning and birth defects view twins as individuals, not twin pairs. Zygosity determination of same-sex twin pairs is rarely performed at birth, and same-sex pairs are often regarded as monozygotic (MZ) twin pairs. This assumption necessarily underestimates the resemblance of MZ pairs according to the proportion of dizygotic (DZ) pairs.

Given these circumstances, ART data present a unique opportunity for twin studies, as most twins after ART are DZ. The first step in genetic epidemiologic analyses is to clarify familial aggregation of targeted traits. To identify familial aggregation, it is important to compare the concordance rate of birth defects in DZ twin pairs (ie, siblings that develop together in the same womb)^2^^,^^3^ with the prevalence of birth defects in the general population. The present study used nationwide data on ART to calculate the concordance rate of twin pairs and examine familial aggregation of birth defects.

## METHODS

### Outline of Japanese ART data

The method for collecting data has been described elsewhere.^4^ Almost all medical institutions that perform ART are registered with the Japan Society of Obstetrics and Gynecology (JSOG). Starting in 2004, an annual list of all ART pregnancies resulting in birth defects has been presented in the JSOG annual ART reports (in Japanese). The author used these case report data from 2004–2009 as initial information. The items included are ART method, blastocyst transfer, maternal age, perinatal outcome and gestational week, plurality, sex, early neonatal infant death up to day 6, and disease name. Within the study period, there were 159 451 singleton pregnancies, 17 258 twin pregnancies, and 839 triplet/+ pregnancies.

Birth defects were reclassified according to the International Classification of Diseases, 10th edition (ICD-10), 2003 version. Diseases that were classified in the categories corresponding to ICD-10 codes Q00–Q99 (ie, congenital malformations, deformations, and chromosomal abnormalities) were selected and analyzed. In total, 1502 abortions, stillbirths, and live births with birth defects were included.

The present author paired twins, using information on birth year, maternal age, gestational weeks, ART method, blastocyst implantation, and plurality. Other information on twin status was also considered; for example, first- and second-born twins were clearly described and listed.

### Statistical analyses

All concordant pairs were listed with their demographic data and neonatal outcome. The pairwise and probandwise concordance rates^5^ were then calculated for each major organ system category, each subcategory, and, in some cases, each disease.

In the present study, the terms “concordant pair” and “discordant pair” are used to describe the disease condition of a given twin pair. The pairwise concordance rate is the probability that both members of a twin pair are affected if at least 1 member of the pair is affected. The probandwise concordance rate is the probability that a twin is affected if his/her co-twin is affected. Only probandwise concordance rates can be directly compared with risk rates reported for other familial pairings and with population prevalence figures.^5^ Pairwise concordance rates were calculated as C/(C + D), and probandwise concordance rates as 2 × C/(2 × C + D), where C denotes the number of affected concordant pairs and D denotes the number of discordant pairs.^5^

Recurrence risk ratios (RRRs)^6^ were used as indicators of familial aggregation of birth defects and were calculated as the ratio of the risk of disease manifestation (which, given that one’s relative is affected, correspond to the probandwise concordance rate of twin pairs in the present study) to the disease prevalence in the general population.

Moreover, the author estimated the recurrence risk of DZ pairs, which have the same genetic resemblance as sib-pairs. According to Edwards,^7^ if a targeted disease is determined by multifactorial inheritance, its frequency in sib-pairs or DZ twin pairs approximates the square root of disease prevalence in the general population. Thus, the present study compared the probandwise concordance rate of any birth defect in twin pairs with the estimated recurrence risk in sib-pairs and DZ pairs.

## RESULTS

From among 247 twins with any birth defect, a total of 236 twin pairs were identified: 11 concordant and 225 discordant pairs. Thus, 1.4% (236/17 258) of twin pairs (pregnancies) had at least 1 affected member.

Demographic and perinatal outcome data for all concordant pairs are presented in Table 1. They comprise 6 male–male, 1 female–female, and 4 opposite-sex pairs. Two of the 11 pairs were stillbirth–stillbirth. The records for 6 of the 9 live-birth pairs showed no early neonatal infant death, although the outcome of the other 3 pairs is not known.

**Table 1.** List of concordant pairs

ID	Classification	Maternal age, y	Gestational weeks	Code	Disease	Sex	Birth status	Early neonatal death	Code	Disease	Sex	Birth status	Early neonatal death	Sex combination
1	Concordance of disease	38	35	Q21 (Q210)	Ventricular septal defect	M	LB	N	Q21 (Q210)	Ventricular septal defect	F	LB	N	OS
									Q21 (Q211)	Atrial septal defect				
									Q25 (Q250)	Patent ductus arteriosus				
2	Concordance of disease	37	26	Q25 (Q250)	Patent ductus arteriosus	M	LB	N	Q25 (Q250)	Patent ductus arteriosus	M	LB	N	MM
3	Concordance of disease	31	27	Q25 (Q250)	Patent ductus arteriosus	M	LB	U	Q25 (Q250)	Patent ductus arteriosus	F	LB	U	OS
									Q32	Congenital malformations of trachea and bronchus				
4	Concordance of disease	35	38	Q22	Congenital malformations of pulmonary and tricuspid valves	F	LB	N	Q42 (Q423)	Congenital absence, atresia and stenosis of anus without fistula	M	LB	N	OS
				Q42 (Q423)	Congenital absence, atresia and stenosis of anus without fistula									
5	Concordance of subcategory	32	37	Q04	Other congenital malformations of brain (lissencephaly)	M	LB	N	Q04	Other congenital malformations of brain (lissencephaly)	M	LB	N	MM
6	Concordance of subcategory	32	37	Q04	Other congenital malformations of brain (lissencephaly)	M	LB	N	Q04	Other congenital malformations of brain (lissencephaly)	M	LB	N	MM
7	Concordance of subcategory	30	17	Q17	Other congenital malformations of ear	M	SB	—	Q17	Other congenital malformations of ear	M	SB	—	MM
8	Concordance of subcategory	35	36	Q37	Cleft palate with cleft lip	M	LB	U	Q37	Cleft palate with cleft lip	M	LB	U	MM
9	Concordance of subcategory	31	21	Q73	Reduction defects of unspecified limb (brachymelia)	M	SB	—	Q73	Reduction defects of unspecified limb (brachymelia)	F	SB	—	OS
10	Concordance of major category	40	38	Q20	Congenital malformations of cardiac chambers and connections	F	LB	U	Q21 (Q210)	Ventricular septal defect	F	LB	U	FF
11	Concordance of major category	29	33	Q25 (Q250)	Patent ductus arteriosus	M	LB	N	Q20	Congenital malformations of cardiac chambers and connections	M	LB	N	MM
									Q21 (Q210)	Ventricular septal defect				
									Q22	Congenital malformations of pulmonary and tricuspid valves				
									Q27	Other congenital malformations of peripheral vascular system				

Order of pairs does not necessarily reflect birth order (ie, first- and second-born).

M: male, F: female; OS: opposite-sex; LB: live birth, SB: stillbirth; N: no, U: unknown.

See Table 2 for more information on classification codes.

The calculated concordance rates and RRRs are shown in Table 2. Regarding classification by major organ system, relatively high probandwise concordance rates were observed for congenital malformations of eye, ear, face, and neck (11.8%), cleft lip and cleft palate (10.5%), congenital malformations of the nervous system (9.8%), and other congenital malformations of the digestive system (9.5%).

**Table 2.** Concordance rates in twin pairs and recurrence risk ratios (RRRs) for birth defects

Major classification code and small disease classification (International Classification of Diseases, 10th edition, 2003 version)			Twin pairs^a,c^				Total		RRR (= A/B)^c^ (95% CI)
	
			C (*n*)	D (*n*)	Concordance rate (%) (95% CI)		*n*	Prevalence (%)^b^ (95% CI) (B)	

					Probandwise (A)	Pairwise			
Q00–Q07		Congenital malformations of the nervous system	2	37	9.8 (0.0, 22.3)	5.1 (0.0, 12.1)	142	0.073 (0.061, 0.085)	133 (52, 343)
	Q00	Anencephaly and similar malformations	0	9	0.0	0.0	49	0.025 (0.018, 0.032)	
	Q01	Encephalocele	0	3	0.0	0.0	7	0.004 (0.001, 0.006)	
	Q02	Microcephaly	0	1	0.0	0.0	3	0.002 (0.000, 0.003)	
	Q03	Congenital hydrocephalus	0	10	0.0	0.0	33	0.017 (0.011, 0.023)	
	Q04	Other congenital malformations of brain	2	3	57.1	40.0	15	0.008 (0.004, 0.012)	
	Q05	Spina bifida	0	10	0.0	0.0	30	0.015 (0.010, 0.021)	
	Q07	Other congenital malformations of nervous system	0	1	0.0	0.0	5	0.003 (0.000, 0.005)	
Q10–Q18		Congenital malformations of eye, ear, face and neck	1	15	11.8 (0.0, 32.8)	6.3 (0.0, 18.1)	98	0.051 (0.041, 0.061)	233 (62, 869)
	Q10	Congenital malformations of eyelid, lacrimal apparatus and orbit	0	3	0.0	0.0	6	0.003 (0.001, 0.006)	
	Q16	Congenital malformations of ear causing impairment of hearing	0	1	0.0	0.0	7	0.004 (0.001, 0.006)	
	Q17	Other congenital malformations of ear	1	10	16.7 (0.0, 45.2)	9.1 (0.0, 26.1)	72	0.037 (0.029, 0.046)	449 (124, 1625)
	Q18	Other congenital malformations of face and neck	0	1	0.0	0.0	3	0.002 (0.000, 0.003)	
Q20–Q28		Congenital malformations of the circulatory system	5	106	8.6 (1.6, 15.7)	4.5 (0.6, 8.4)	560	0.289 (0.265, 0.313)	30 (16, 54)
	Excluding patent ductus arteriosus		3	86	6.5 (0.0, 13.5)	3.4 (0.0, 7.1)	499	0.257 (0.235, 0.280)	25 (12, 55)
	Q20	Congenital malformations of cardiac chambers and connections	0	5	0.0	0.0	32	0.016 (0.011, 0.022)	
	Q21	Congenital malformations of cardiac septa	1	55	3.5 (0.0, 10.2)	1.8 (0.0, 5.3)	315	0.162 (0.144, 0.180)	22 (6, 85)
		Q210 Ventricular septal defect	1	38	5.0 (0.0, 14.4)	2.6 (0.0, 7.5)	214	0.110 (0.096, 0.125)	45 (12, 176)
		Q211 Atrial septal defect	0	9	0.0	0.0	66	0.034 (0.026, 0.042)	
		Q213 Tetralogy of Fallot	0	8	0.0	0.0	30	0.015 (0.010, 0.021)	
	Q22	Congenital malformations of pulmonary and tricuspid valves	0	8	0.0	0.0	33	0.017 (0.011, 0.023)	
	Q23	Congenital malformations of aortic and mitral valves	0	1	0.0	0.0	15	0.008 (0.004, 0.012)	
	Q24	Other congenital malformations of heart	0	3	0.0	0.0	38	0.020 (0.013, 0.026)	
	Q25	Congenital malformations of great arteries	2	29	12.1 (0.0, 27.4)	6.5 (0.0, 15.1)	100	0.052 (0.041, 0.062)	235 (92, 601)
		Q250 Patent ductus arteriosus	2	20	16.7 (0.0, 36.9)	9.1 (0.0, 21.1)	61	0.031 (0.024, 0.039)	530 (209, 1342)
	Q26	Congenital malformations of great veins	0	2	0.0	0.0	13	0.007 (0.003, 0.010)	
	Q27	Other congenital malformations of peripheral vascular system	0	3	0.0	0.0	9	0.005 (0.002, 0.008)	
Q30–Q34		Congenital malformations of the respiratory system	0	4	0.0	0.0	23	0.012 (0.007, 0.017)	
	Q32	Congenital malformations of trachea and bronchus	0	1	0.0	0.0	3	0.002 (0.000, 0.003)	
	Q33	Congenital malformations of lung	0	3	0.0	0.0	12	0.006 (0.003, 0.010)	
Q35–Q37		Cleft lip and cleft palate	1	17	10.5 (0.0, 29.5)	5.6 (0.0, 16.1)	98	0.051 (0.041, 0.061)	208 (55, 784)
	Q35	Cleft palate	0	3	0.0	0.0	26	0.013 (0.008, 0.019)	
	Q36	Cleft lip	0	3	0.0	0.0	23	0.012 (0.007, 0.017)	
	Q37	Cleft palate with cleft lip	1	11	15.4 (0.0, 42.0)	8.3 (0.0, 24.0)	49	0.025 (0.018, 0.032)	609 (165, 2246)
Q38–Q45		Other congenital malformations of the digestive system	1	19	9.5 (0.0, 26.9)	5.0 (0.0, 14.6)	135	0.070 (0.058, 0.081)	137 (36, 517)
	Q39	Congenital malformations of esophagus	0	5	0.0	0.0	28	0.014 (0.009, 0.020)	
	Q40	Other congenital malformations of upper alimentary tract	0	2	0.0	0.0	8	0.004 (0.001, 0.007)	
	Q41	Congenital absence, atresia and stenosis of small intestine	0	3	0.0	0.0	20	0.010 (0.006, 0.015)	
	Q42	Congenital absence, atresia and stenosis of large intestine	1	5	28.6	16.7	41	0.021 (0.015, 0.028)	
		Q423 Congenital absence, atresia and stenosis of anus without fistula	1	5	28.6	16.7	41	0.021 (0.015, 0.028)	
	Q43	Other congenital malformations of intestine	0	2	0.0	0.0	24	0.012 (0.007, 0.017)	
	Q44	Congenital malformations of gallbladder, bile ducts and liver	0	2	0.0	0.0	10	0.005 (0.002, 0.008)	
Q50–Q56		Congenital malformations of genital organs	0	12	0.0	0.0	59	0.030 (0.023, 0.038)	
	Q53	Undescended testicle	0	3	0.0	0.0	23	0.012 (0.007, 0.017)	
	Q54	Hypospadias	0	8	0.0	0.0	31	0.016 (0.010, 0.022)	
	Q55	Other congenital malformations of male genital organs	0	1	0.0	0.0	4	0.002 (0.000, 0.004)	
Q60–Q64		Congenital malformations of the urinary system	0	6	0.0	0.0	64	0.033 (0.025, 0.041)	
	Q60	Renal agenesis and other reduction defects of kidney	0	1	0.0	0.0	14	0.007 (0.003, 0.011)	
	Q61	Cystic kidney disease	0	2	0.0	0.0	10	0.005 (0.002, 0.008)	
	Q62	Congenital obstructive defects of renal pelvis and congenital malformations of ureter	0	1	0.0	0.0	28	0.014 (0.009, 0.020)	
	Q64	Other congenital malformations of urinary system	0	2	0.0	0.0	9	0.005 (0.002, 0.008)	
Q65–Q79		Congenital malformations and deformations of the musculoskeletal system	1	36	5.3 (0.0, 15.2)	2.7 (0.0, 7.9)	268	0.138 (0.122, 0.155)	38 (10, 148)
	Q65	Congenital deformities of hip	0	1	0.0	0.0	10	0.005 (0.002, 0.008)	
	Q66	Congenital deformities of feet	0	3	0.0	0.0	28	0.014 (0.009, 0.020)	
		Q668 Other congenital deformities of feet	0	3	0.0	0.0	24	0.012 (0.007, 0.017)	
	Q68	Other congenital musculoskeletal deformities	0	1	0.0	0.0	15	0.008 (0.004, 0.012)	
	Q69	Polydactyly	0	9	0.0	0.0	63	0.032 (0.024, 0.040)	
	Q70	Syndactyly	0	3	0.0	0.0	31	0.016 (0.010, 0.022)	
	Q71	Reduction defects of upper limb	0	2	0.0	0.0	11	0.006 (0.002, 0.009)	
	Q73	Reduction defects of unspecified limb	1	1	66.7	50.0	6	0.003 (0.001, 0.006)	
	Q74	Other congenital malformations of limb(s)	0	1	0.0	0.0	9	0.005 (0.002, 0.008)	
	Q75	Other congenital malformations of skull and face bones	0	2	0.0	0.0	12	0.006 (0.003, 0.010)	
	Q77	Osteochondrodysplasia with defects of growth of tubular bones and spine	0	1	0.0	0.0	7	0.004 (0.001, 0.006)	
	Q79	Congenital malformations of the musculoskeletal system, not elsewhere classified	0	12	0.0	0.0	55	0.028 (0.021, 0.036)	
		Q790 Congenital diaphragmatic hernia	0	4	0.0	0.0	20	0.010 (0.006, 0.015)	
		Q792 Exomphalos	0	5	0.0	0.0	19	0.010 (0.005, 0.014)	
Q80–Q89		Other congenital malformations	0	14	0.0	0.0	68	0.035 (0.027, 0.043)	
	Q82	Other congenital malformations of skin	0	2	0.0	0.0	20	0.010 (0.006, 0.015)	
	Q85	Phakomatoses, not elsewhere classified	0	1	0.0	0.0	2	0.001 (0.000, 0.002)	
	Q87	Other specified congenital malformation syndromes affecting multiple systems	0	8	0.0	0.0	20	0.010 (0.006, 0.015)	
	Q89	Other congenital malformations, not elsewhere classified	0	3	0.0	0.0	25	0.013 (0.008, 0.018)	
		Q897 Multiple congenital malformations, not elsewhere classified	0	1	0.0	0.0	11	0.006 (0.002, 0.009)	
Q90–Q99		Chromosomal abnormalities, not elsewhere classified	0	27	0.0	0.0	288	0.148 (0.131, 0.166)	
	Q90	Down’s syndrome	0	19	0.0	0.0	178	0.092 (0.078, 0.105)	
	Q91	Edwards’ syndrome and Patau’s syndrome	0	8	0.0	0.0	72	0.037 (0.029, 0.046)	
		Q913 Edwards’ syndrome, unspecified	0	5	0.0	0.0	57	0.029 (0.022, 0.037)	
		Q917 Patau’s syndrome, unspecified	0	3	0.0	0.0	15	0.008 (0.004, 0.012)	

Any birth defects			11	225	8.9 (4.0, 13.8)	4.7 (2.0, 7.4)	1493	0.770 (0.731, 0.809)	12 (8, 17)

Excluding patent ductus arteriosus			9	205	8.1 (3.1, 13.0)	4.2 (1.5, 6.9)	1432	0.738 (0.700, 0.776)	11 (7, 17)

Singleton pregnancies = 159 451; twin pregnancies = 17 258; total fetuses/neonates = 193 967.

C: concordant twin pair, D: discordant twin pair.

^a^Only twin pairs with at least 1 affected member are listed.

^b^Total prevalence was calculated per fetuses/neonates.

^c^Concordance rates and RRRs with their 95% CI were calculated for disease classifications that had >10 total concordant/discordant twin pairs and a concordance rate not equal to 0.

Among disease classifications with more than 10 total concordant/discordant twin pairs, RRRs were greater than 200 for congenital malformations of eye, ear, face, and neck (RRR = 233), specifically other congenital malformations of ear (RRR = 449); congenital malformations of the great arteries (RRR = 235), specifically those of the patent ductus arteriosus (RRR = 530); and cleft lip and cleft palate (RRR = 208), specifically cleft palate with cleft lip (RRR = 609).

The probandwise concordance rate of any birth defect was 8.9%, which was nearly identical to the estimated recurrence risk among sib-pairs and DZ pairs, namely, 8.8% (the square root of 0.77, see Table 2).

## DISCUSSION

### Method of analysis

Correct zygosity diagnosis is rare in most studies of birth defects. Researchers have often compared resemblance between same-sex pairs (as a proxy for MZ pairs) and opposite-sex DZ pairs, on the assumption that there is no sex difference in prevalence. In the present study, information was obtained only from probands. In such a situation, the probandwise concordance rate is the second-best measure of resemblance in twin pairs.

Although most subjects in the present study were DZ pairs, some MZ pairs may well have also been included. According to a recent meta-analysis by Vitthala et al,^8^ the incidence of MZ twins after ART is 2.25 times that after natural conception. Caution is warranted in interpreting these values because most previous studies used the pairwise rather than the probandwise concordance rate. Ascertainment bias in the identification of twin pairs would be small in the present sample, since birth defects during pregnancy or the neonatal period (at the latest) were reported in the same hospital. However, this ascertainment period may underestimate the concordance rate if pairs differed in the age when symptoms of birth defects became obvious.

### Birth defects in twin pairs

The number of concordant pairs was clearly higher for male–male pairs than for female–female and opposite-sex pairs. No previous study reported that concordance rates differed according to the sex combination of twin pairs.

The concordance rates of birth defects in twin pairs, as determined in previous large studies, are shown in Table 3. The concordance rate for any birth defect is higher in MZ pairs and all twin pairs as compared with DZ pairs and opposite-sex DZ pairs,^9^^–^^12^ respectively, which suggests a genetic contribution to birth defects. The probandwise concordance rate of any birth defect (8.9%) was nearly identical to the estimated recurrence risk among sib-pairs (8.8%) and much higher than the prevalence in the general population (0.77%). These results suggest familial aggregation of birth defects and that the origin of this aggregation is multifactorial inheritance.

**Table 3.** Concordance rates of birth defects in previously published twin studies

Birth defects		Author(s)	Year of data collection	No. of pairs	Data source	Zygosity	Twin pairs		Concordance rate (%)	
	
							Concordant (*n* pairs)	Discordant (*n* pairs)	Probandwise	Pairwise
Any birth defect		Myrianthopoulos [1976]^9^	Not mentioned	615	Collaborative perinatal	MZ^a^	23	46	50.0	33.3
					project	DZ^a^	6	79	13.2	7.1
		Imaizumi et al [1990]^10^	1974	12 392	Population-based	All	22	34	56.4	39.3
		Kato & Fujiki [1992]^11^	1979–1990	968	Hospital-based	All	3	39	13.3	7.1
						OS	0	8	0.0	0.0
		Campana & Roubicek [1996]^12^	1982–1995	690	Hospital-based	All	5	25	28.6	16.7

Congenital malformations of the nervous system										
	neural tube defects	Janerich & Piper [1978]^23^	1961–1974	23 310	Population-based	All	4	55	12.7	6.8
	anencephalus	Imaizumi [1978]^21^	1969–1976	Not mentioned	Death certificate	All	9	100	15.3	8.3
	neural tube defects	Windham & Sever [1982]^24^	1966–1972	8 440	Population-based	All	1	26	7.1	3.7
	hydrocephalus	Imaizumi [1989]^22^	1969–1985	Not mentioned	Death certificate	All	16	91	26.0	15.0
	anencephalus	Imaizumi et al [1990]^10^	1974	12 392	Population-based	All	4	8	50.0	33.3
	hydrocephalus	Imaizumi et al [1990]^10^	1974	12 392	Population-based	All	3	10	37.5	23.1
	anencephalus	Campana & Roubicek [1996]^12^	1982–1995	690	Hospital-based	All	4	27	22.9	12.9
	neural tube defects	Deak et al [2008]^25^	1993–2007	47	Many data sources	MZ^b^	2	3	57.1	40.0
						DZ^b^	3	32	15.8	8.6

Congenital malformations of the circulatory system										
	same congenital heart disease	Kenna et al [1975]^13^	1960–1969	Not mentioned	Population-based	MZ^c^	2	13	23.5	13.3
	any congenital heart disease	Kenna et al [1975]^13^	1960–1970	Not mentioned	Population-based	DZ^c^	2	10	28.6	16.7
	patent ductus arteriosus	Layde et al [1980]^19^	1969–1976	1 670 twins	Population-based	All	7	14	50.0	33.3
	congenital heart disease	Caputo et al [2005]^2^	1999–2002	66	Patient enrollment	DZ^d^	9	57	24.0	13.6
	cardiovascular defects	Hardin et al [2009]^14^	1983–2003	56 709	Birth defect monitoring	All	331	2 404	21.6	12.1
						OS	53	650	14.0	7.5
	ventricular septal defect	Hardin et al [2009]^14^	1983–2003	56 709	Birth defect monitoring	All	4	110	6.8	3.5
						MZ^e^	4	87	8.4	4.4
						DZ^e^	0	23	0.0	0.0

Cleft lip and cleft palate										
	cleft lip/palate	Lin et al [1999]^26^	1977–1997	38	Hospital-based	MZ^f^	4	3	72.7	57.1
						DZ^f^	1	10	16.7	9.1
	cleft lip with/without cleft palate	Grosen et al [2011]^3^	1936–2004	130 710	Population-based	MZ^g^	8	16	50.0	33.3
						DZ^g^	4	93	7.9	4.1
	cleft palate	Grosen et al [2011]^3^	1936–2004	130 710	Population-based	MZ^g^	1	4	33.3	20.0
						DZ^g^	1	25	7.4	3.8

Other congenital malformations of the digestive system										
	esophageal atresia	David & O’Callaghan [1975]^27^	1942–1973	19	Hospital-based	All	2	17	19.0	10.5
	esophageal atresia	Orford et al [2000]^28^	1948–1998	51	Hospital-based	All	2	47	7.8	4.1

Congenital malformations and deformations of the musculoskeletal system										
	Bochdalek diaphragmatic hernia	Pober et al [2005]^30^	1972–1974, 1979–2003	8	Hospital-based	All	0	8	0.0	0.0

Most probandwise concordance rates were recalculated by using the number of concordant and discordant pairs in the literature.

MZ: monozygotic, DZ: dizygotic, OS: opposite-sex

^a^blood type and placenta, ^b^maternal report, ^c^placentation and chorionicity, ^d^blood type, chorionicity, physical characteristics, ^e^placenta and reported type, ^f^blood type, physical resemblance, chorionicity, ^g^questionnaire.

The probandwise concordance rate of congenital malformations of the circulatory system was 30-fold higher than the prevalence in the general population. Kenna et al^13^ found 2 concordant pairs out of 15 MZ pairs and 2 concordant pairs with different heart lesions out of DZ 12 pairs. According to Hardin et al,^14^ the probandwise concordance rate for opposite-sex DZ twin pairs was 14.0%. A small number of studies directly compared the recurrence risk of twin pairs with that of first-degree relatives. Caputo et al^2^ compared recurrence risk in DZ pairs and sib-pairs and concluded that the higher recurrence and concordance of congenital heart disease in DZ twins might depend on a poorly understood environmental risk during pregnancy. However, Øyen et al^15^ found that intrauterine conditions had no effect, after comparing the RRRs of opposite-sex twin pairs and first-degree relatives.

It has been suggested that patent ductus arteriosus (PDA) is related to prematurity and is consequently more prevalent in twins.^16^^–^^18^ Layde et al^19^ found that a high concordance rate was observed in same-sex pairs, which suggests both a strong genetic component to the etiology of PDA and high rates of prematurity in twin pairs. The present finding of a high concordance rate and RRR for PDA supports the genetic/shared environmental hypothesis. When concordance rates were calculated after excluding PDA cases, the results were not markedly different, as shown in Table 2.

Regarding congenital malformations of the nervous system in twins, neural tube defects have been well examined.^10^^,^^12^^,^^20^^–^^25^ The present study observed no concordant pair with anencephalus, spina bifida, or hydrocephalus. There were 2 male–male concordant pairs with lissencephaly (subcategory Q04), but no other such cases among twins or singletons, suggesting that the original data were incorrectly input.

One male–male concordant pair showed both micrognathia and low-set ear (subcategory Q17). There is no population-based twin study of these defects.

There was one male–male concordant pair who had cleft palate with cleft lip (subcategory Q37), with a 15.4% probandwise concordance rate. This value is similar to that of DZ pairs (16.7%), as reported by Lin et al.^26^ According to Grosen et al,^3^ the probandwise concordance rate for cleft lip/cleft palate was higher for MZ pairs than for DZ pairs. However, they also found that the recurrence risk for both types of clefts was greater in DZ twins than in non-twin siblings, suggesting intrauterine environmental effects on these traits. The fact that the RRR for cleft palate with cleft lip was highest in the present study supports their results.

There was no concordant pair with esophageal atresia (subcategory Q39). David and O’Callaghan^27^ found that the probandwise concordance rate for this condition was 19.0%, although Orford et al^28^ reported a low pairwise concordance rate (4.1%). There was 1 concordant opposite-sex DZ pair of imperforate anus (subcategory Q42). Källén^17^ reported that for alimentary atresia, including imperforate anus, concordance was rather common among same-sex pairs.

There was 1 concordant opposite-sex DZ pair with brachymelia (subcategory Q73). Métneki et al^29^ studied the occurrence of congenital limb reduction defects in twins and concluded that genetic factors have a limited role in pathogenesis. Pober et al^30^ conducted a large twin study of Bochdalek diaphragmatic hernia, including 8 twin cases with no concordant pairs. The concordance rate of congenital malformations and deformations of the musculoskeletal system was low in the present study, in contrast to the relatively high prevalence among the general population.

### Limitations

Most limitations of this study are due to deficiencies in the data collection system. The first limitation was the lack of a zygosity classification for same-sex pairs. Second, pairing of twins was not necessarily complete. Some concordant pairs might not have been real twin pairs. Third, the statistical power of the analyses was limited. The present concordance rates might be strongly influenced by chance factors, since most disease concordance rates were calculated on the basis of no or 1 concordant pair.

### Conclusions

The present results provide a good overview of concordance rates among twin pairs with birth defects after ART. Strong familial aggregation was observed for some birth defects.

## ONLINE ONLY MATERIALS

The Japanese-language abstract for articles can be accessed by clicking on the tab labeled Supplementary materials at the journal website http://dx.doi.org/10.2188/jea.JE20120103.

Abstract in Japanese.
