# Supplementary material for: Concordance Rates of Birth Defects After Assisted Reproductive Technology Among 17 258 Japanese Twin Pregnancies: A Nationwide Survey, 2004–2009
Source: J Epidemiol. 2013 Jan 5;23(1):63–9. doi: 10.2188/jea.JE20120103 (PMC3700233; doi:10.2188/jea.JE20120103)
Supplement: Abstract in Japanese. [file je-23-063-s001.pdf]

生殖補助医療による 17258 件のふたご妊娠における先天異常の一致率：2004 年から 2009 年の全国的調査

大木秀一
石川県立看護大学健康科学講座

【背景】生殖補助医療によるふたごの大部分は二卵性である。二卵性ふたごペアの分析は、先天異常の形成過程における家族集積性を検討するうえで有効である。

【方法】日本産科婦人科学会が公表している全国的な生殖補助医療データを利用して、家族集積性の指標として再発危険率（RRR）を算出した。再発危険率はふたごペアにおける先天異常の発端者一致率を一般集団における先天異常の発生頻度で除した値と定義した。国際疾病分類第 10 版（ICD-10）のコード Q00-Q99（先天奇形、変形及び染色体異常）に従って、先天異常の症例データを再分類した。2004 年から 2009 年までに 17258 件のふたご妊娠があった。

【結果】236 組のペアに少なくとも 1 児の先天異常が認められた。一致が 11 組、不一致 225 組であった。主要組織分類に関しては、眼・耳・顔面及び頚部の先天奇形（11.8%）、唇裂及び口蓋裂（10.5%）、神経系の先天奇形（9.8%）、消化器系のその他の先天奇形（9.5%）で高い発端者一致率が認められた。眼・耳・顔面及び頚部の先天奇形（RRR=233）、特に、耳のその他の先天奇形（RRR=449）、大型動脈の先天奇形（RRR=235）、特に、動脈管開存症（RRR=530）、唇裂及び口蓋裂（RRR=208）、特に、唇裂を伴う口蓋裂（RRR=609）で高い再発危険率を認めた。先天異常全体の発端者一致率（8.9%）は、多因子遺伝を仮定して推定した同胞再発危険率（8.8%）とほぼ一致した。

【結論】今回の知見は、ある種の先天異常に家族集積性があることを示唆する。

キーワード：先天異常、生殖補助医療、ふたごペア、一致率、全国的疫学研究
